# Exploring sex hormones as potential prognostic biomarkers in prolonged disorders of consciousness: a sex-specific retrospective study

**DOI:** 10.3389/fneur.2026.1878434

**Published:** 2026-09-03

**Authors:** Dandan Xia, Hui Feng, Yuqing Han, Lizhi Liu, Fangyu Chen, Juanjuan Fu

**Affiliations:** Department of Rehabilitation Medicine, The Affiliated Jiangning Hospital of Nanjing Medical University, Nanjing, China

**Keywords:** coma recovery scale-revised score, disorders of consciousness, prognosis, prolactin, sex hormones, testosterone

## Abstract

**Background:**

Patients with prolonged disorders of consciousness (pDoC) present significant clinical challenges, directly influencing awakening strategies and the allocation of healthcare resources. This study aimed to evaluate serum sex hormone levels in pDoC patients and assess their potential for predicting clinical outcomes.

**Methods:**

This retrospective study initially screened 142 patients with prolonged disorders of consciousness (pDoC) admitted between January 2023 and January 2025. After applying exclusion criteria, a final cohort of 119 patients (77 males and 42 females) was included for analysis. Within 1 week of enrollment, the Chinese Coma Recovery Scale-Revised (CRS-R) score and serum sex hormone levels were assessed. Outcomes were evaluated at 6 months using the Glasgow Outcome Scale–Extended (GOS-E) and classified as good outcome(GOSE score≥4) or poor outcome(GOSE score <4). Statistical analyses were performed to explore the association between serum sex hormone levels and GOSE scores to identify potential prognostic markers.

**Results:**

Among male patients, the good outcome group showed higher testosterone levels (*p* = 0.02) and a trend toward lower prolactin levels (*p* = 0.06) compared to the poor outcome group. In female patients, the good outcome group had lower levels of prolactin, follicle-stimulating hormone, and luteinizing hormone (*p* < 0.05). However, after Bonferroni correction for multiple comparisons, none of these associations remained statistically significant. In multivariate logistic regression adjusting for age, disease duration, and etiology, log-transformed testosterone showed a consistent association with favorable outcomes in males (odds ratio, OR = 2.97, 95% CI: 1.29–7.70, *p* = 0.02), though this did not survive Bonferroni correction (P_bonf = 0.10). Prolactin and other sex hormones were not independent predictors in either sex. Receiver operating characteristic (ROC) analysis demonstrated that testosterone alone yielded an area under the curve (AUC) of 0.656 (95% CI: 0.532–0.781), with minimal improvement after etiology adjustment (AUC = 0.691, 95% CI: 0.573–0.809; *p* = 0.45).

**Conclusion:**

Serum testosterone showed exploratory, hypothesis-generating associations with favorable outcomes in male pDoC patients. However, these findings did not survive correction for multiple testing, and the predictive performance was modest. Sex hormone levels did not demonstrate clear prognostic value in female patients. These preliminary observations underscore the need for larger, prospective studies with standardized endocrine protocols to validate the potential role of sex hormones as prognostic biomarkers in pDoC.

## Introduction

Consciousness is regulated by a complex network of brain structures (1, 2), with the ascending reticular activating system (ARAS) playing a central role in maintaining wakefulness. Disorders of consciousness (DoC) following severe brain injury—whether traumatic or non-traumatic—are often caused by transient dysfunction or structural damage to the ARAS (3, 4). This network links brainstem reticular formations with thalamic nuclei, the basal forebrain, hypothalamus, and cerebral cortex (1). While neuroinflammation, seizures, and metabolic disturbances can influence consciousness (5, 6), the exact mechanisms underlying DoC remain unclear.

The clinical assessment of consciousness remains controversial. Behavioral assessments such as the Coma Recovery Scale-Revised (CRS-R) score have a misdiagnosis rate as high as 40% (7, 8). Other behavioral tools commonly used in clinical practice include the Sensory Modality Assessment and Rehabilitation Technique (SMART) (9, 10), the Wessex Head Injury Matrix (WHIM) (11), the Disorders of Consciousness Scale (DOCS) (12, 13), and the Simplified Evaluation of Consciousness Disorders (SECONDs) (14). However, all behavioral assessments are susceptible to patient-related factors (e.g., motor deficits, aphasia, or fluctuating arousal) and environmental biases, which limit their diagnostic accuracy. Aside from somatosensory evoked potentials, which offer predictive value in hypoxic–ischemic encephalopathy (15), no reliable biomarkers for consciousness recovery have been established. This uncertainty may result in inappropriate allocation of rehabilitation resources for patients mistakenly considered to have poor prognoses (16). Thus, identifying biomarkers that can predict outcomes in prolonged disorders of consciousness (pDoC) is essential.

Pituitary dysfunction is a well-known complication after brain injury,often affecting one or more pituitary-target gland axes. Other common complications following brain injury include post-traumatic epilepsy, hydrocephalus, spasticity, cognitive impairment, and neuroendocrine disturbances such as hypothalamic–pituitary–adrenal (HPA) axis dysregulation and central diabetes insipidus (17, 18). These hormonal imbalances are thought to contribute to post-traumatic recovery (19, 20). Although some patients recover partially,the degree of recovery varies widely. The influence of sex differences on outcomes remains debated. In cases of mild traumatic brain injury (TBI), female patients tend to have worse outcomes, and post-traumatic complications disproportionately affect women. Paradoxically, both female sex and postmenopausal status have been reported as risk and protective factors (21–23). Experimental TBI models suggest that female gonadal hormones may have neuroprotective effects (24). Despite increased attention to hormone deficiencies after brain injury, studies on sex hormones as predictors of consciousness recovery are limited. This is particularly true in pDoC, where the prognostic value of sex hormones is unclear. This study aimed to investigate whether serum sex hormone levels could serve as biomarkers for predicting neurological recovery in pDoC patients, supporting clinical decisions and guiding resource allocation.

## Methods

### Patients and study design

This retrospective study initially screened 142 patients with pDoC admitted to the Department of Critical Care Rehabilitation at Nanjing Jiangning Hospital from January 2023 to January 2025. After applying exclusion criteria—7 lost to follow-up, 6 with incomplete hormone data, 5 with untreated thyroid disease, 3 receiving hormone replacement therapy, and 2 with neurodegenerative disease—119 patients (77 males and 42 females) were included in the final analysis. Inclusion criteria:age 18–75 years; disease duration of 28–90 days (≥ 28 days consistent with the definition of prolonged disorders of consciousness, and ≤ 90 days ensuring endpoint assessment at 6 months is meaningful and minimizing confounding from time-dependent hormonal fluctuations); first-ever stroke, traumatic brain injury, or hypoxic–ischemic encephalopathy(confirmed by International Classification of Diseases, Tenth Revision (ICD-10) codes and brain computed tomography (CT)); Diagnosis of vegetative state/unresponsive wakefulness syndrome (VS/UWS) or minimally conscious state (MCS) confirmed by via five consecutive weekly Chinese CRS-R score assessments (25, 26); no use of sedatives, anesthetics, or muscle relaxants during evaluation; and written informed consent obtained from legal guardians. Exclusion criteria included: history of previous acute brain injury, neurodegenerative diseases, or peripheral neuropathy; clinically unstable conditions (e.g., hemodynamic instability, severe respiratory failure, or acute hydrocephalus); comorbid malignancies, ongoing hormone suppression therapy, or untreated thyroid disease.

### Data collection and clinical assessment

Demographic and clinical data were collected for all participants, including age, sex, menopausal status, smoking history, alcohol consumption, medical history, and decompressive craniectomy status. Menopausal status was determined based on reports from family members when available; if unavailable, female patients older than 50 years were classified as postmenopausal. Initial clinical assessments were conducted by neurologists with experience in pDoC evaluation. Each patient underwent a comprehensive evaluation over five consecutive days during the first week of admission. All patients received standardized neurological examinations and were assessed using the Chinese CRS-R score (25). The CRS-R score consists of 23 hierarchically organized items across six subscales assessing auditory, visual, motor, verbal/oral, communication, and arousal functions. It is considered the most reliable tool for evaluating consciousness in pDoC and allows differential diagnosis between VS/UWS and MCS. The total CRS-R score ranges from 0 (coma) to 23 (emergence from MCS).

### Serum sex hormone measurements

Serum sex hormone levels were measured during the first week of hospitalization. For female participants, blood samples were collected during either the follicular phase (days 5–10) or luteal phase (days 18–23) of the menstrual cycle. All biochemical analyses were conducted at the Clinical Laboratory of Nanjing Jiangning Hospital using standardized procedures. Estradiol, progesterone, and testosterone levels were measured via radioimmunoassay (Coat-A-Count kits), while follicle-stimulating hormone (FSH), luteinizing hormone (LH), and prolactin levels were determined using electrochemiluminescence immunoassay (Roche Cobas e170 analyzer). All assays were conducted under strict quality control to ensure accurate quantification and account for physiological variations in female hormone levels.

### Definition of outcome

The primary outcome was the patient’s functional outcome assessed by the Glasgow Outcome Scale–Extended (GOS-E) (27), an eight-point scale ranging from1 (death) to 8 (upper good recovery). For the purpose of statistical analysis, the GOS-E was dichotomized as a good neurologic outcome (GOS-E score≥4) and an poor outcome (GOS-E score <4) (28). The secondary outcome is consciousness recovery, measured by the CRS-R score improvement (change from baseline to 6 months). A structured telephone interview was conducted for 6 months follow-up by a trained neurologist who was blinded to the clinical data.

### Statistical analysis

Data normality was assessed using the Shapiro–Wilk test. Continuous variables with normal distribution were expressed as mean ± SD and compared using independent t-tests; non-normally distributed variables were presented as median (IQR) and analyzed with Mann–Whitney U tests. For multivariate logistic regression, all hormone variables were natural log-transformed to stabilize variance. Categorical variables were compared using χ^2^ or Fisher‘s exact tests, as appropriate.

Patients were stratified by GOSE scores into good (≥4) and poor (<4) outcome groups. Multivariate logistic regression was performed to identify independent predictors, with adjustment for age, disease duration, and etiology. Results are reported as adjusted ORs with 95% CIs. ROC curve analysis was used to evaluate predictive performance, with AUC calculations. Correlations between continuous variables were assessed using Spearman’s rank correlation. For multiple comparisons, Bonferroni correction was applied to univariate analyses and multivariate models.

A post-hoc power analysis was performed for the primary finding (testosterone and favorable outcome in males). Based on the observed OR of 2.97 for log-transformed testosterone (≈ OR 1.16 per 1 nmol/L increase in raw testosterone), with *n* = 77 males (44 good, 33 poor outcomes), *α* = 0.05, and event proportion of 0.43, the achieved power was approximately 68% for the log-transformed scale and 60% for raw testosterone. These findings indicate adequate power to detect moderate-to-large effects but limited power for smaller effects, consistent with the exploratory nature of the study. Calculations were performed using G*Power 3.1 (29), assuming 6 predictors and R^2^ = 0.10. All statistical analyses were performed using SPSS version 21.0 (IBM Corp., Armonk, NY, USA) with a two-sided significance level set at *p* < 0.05.

## Results

### Enrollment and characteristics of the pDoC patients

**Figure 1:**
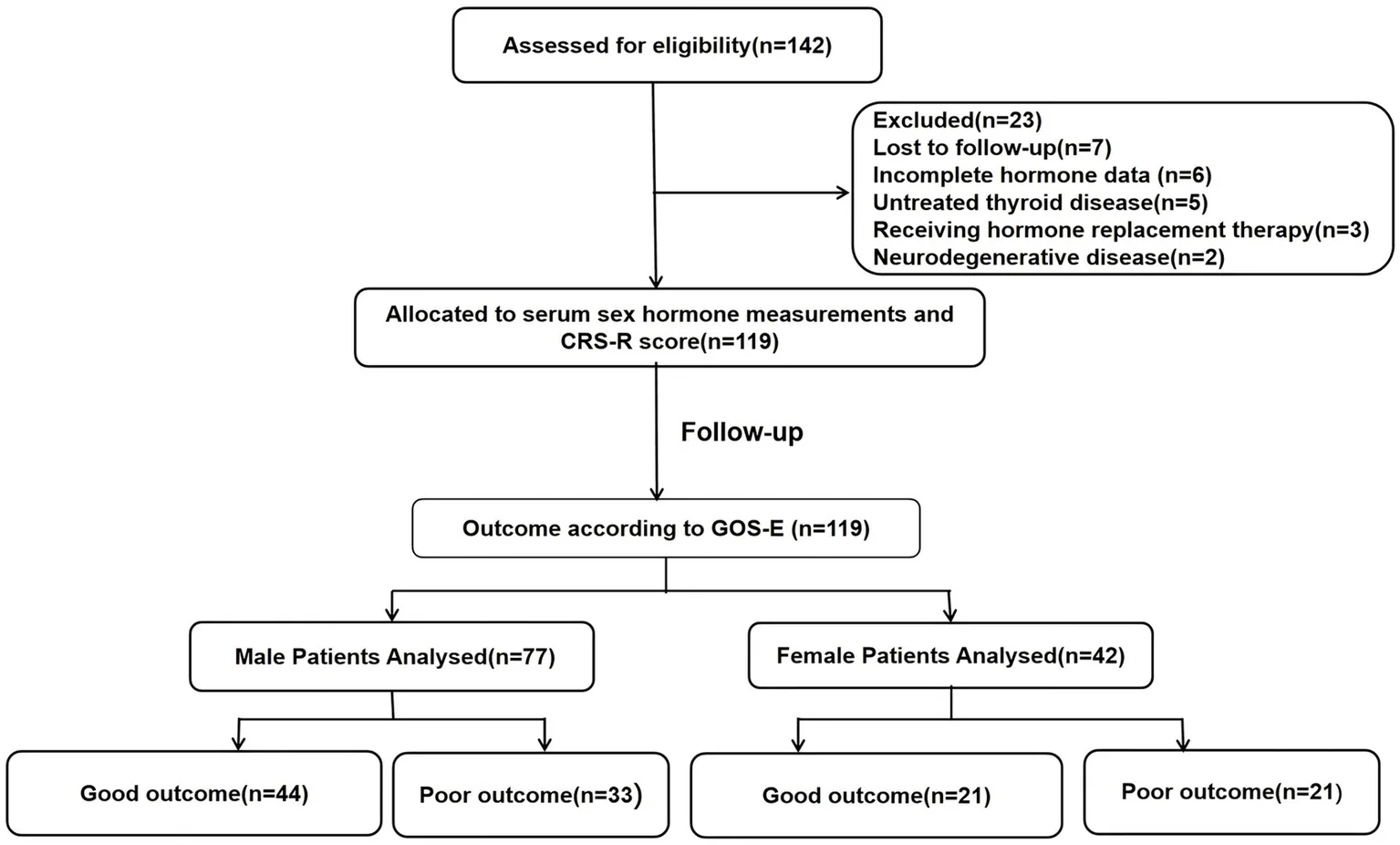
Flow diagram for patient inclusion and exclusion.

The patient screening and enrollment process is summarized in Figure 1. Among the 119 patients included in the final analysis, 77 were male (44 with good outcomes, 33 with poor outcomes) and 42 were female (21 with good outcomes, 21 with poor outcomes).

After stratifying the cohort by sex and analyzing functional outcomes at the 6-month follow-up, no significant differences were observed between male and female subgroups in baseline characteristics, including age, decompressive craniectomy status, medical history, or comorbidities (*p* > 0.05). As expected, the distribution of consciousness states differed significantly between outcome groups. The poor outcome group had a significantly higher proportion of VS/UWS patients compared to MCS patients (*p* < 0.001). In addition, the poor outcome group showed significantly lower baseline CRS-R scores (*p* < 0.001), lower CRS-R scores at the 6-month follow-up (*p* < 0.001), and smaller improvements in CRS-R scores (*p* < 0.001) compared with the good outcome group (Table 1).

**Table 1:** Baseline characteristics of included patients.

Variable		Male			Female		
		Poor outcome (*n* = 33)	Good outcome (*n* = 44)	*p* value	Poor outcome (*n* = 21)	Good outcome (*n* = 21)	*p* value
Mean age in years		56.25 ± 12.92	57.47 ± 12.69	**0.67**	61.14 ± 12.63	63.57 ± 7.93	0.46
Disease duration(days)		55.06 ± 24.78	52.29 ± 25.36	**0.47**	56.62 ± 34.52	62.67 ± 30.37	0.26
ICU stay(days)		22.72 ± 9.71	21.93 ± 10.51	**0.73**	25.14 ± 8.91	22.62 ± 11.39	0.43
Etiology, n(%)	ICH	15(45.5%)	13(29.5%)	**0.14**	13(61.9%)	14(66.7%)	0.42
	TBI	12(36.4%)	26(59.1%)		2(9.5%)	6(28.6%)	
	HIE	6(18.2%)	5(11.4%)		6(28.6%)	1(4.8%)	
Craniectomy, n(%)	N0	20(62.5%)	26(47.3%)	**0.17**	13(61.9%)	9(42.9%)	0.22
	YES	12(37.5%)	29(52.7%)		8(38.1%)	12(57.1%)	
Smoking history, n(%)	N0	5(15.6%)	8(14.5%)	**0.89**	21(100%)	21(100%)	1
	YES	27(84.4%)	47(85.5%)		0(0%)	0(0%)	
Alcohol drinking history, n(%)	N0	6(18.8%)	9(16.4%)	**0.78**	21(100%)	21(100%)	1
	YES	26(81.3%)	46(83.6%)		0(0%)	0(0%)	
Hypertension, n(%)	N0	4(12.5%)	10(18.2%)	**0.49**	6(28.6%)	5(23.8%)	0.73
	YES	28(87.5%)	45(81.8%)		15(71.4%)	16(76.2%)	
Diabetes, n(%)	N0	26(81.3%)	46(83.6%)	**0.78**	17(81%)	14(66.7%)	0.29
	YES	6(18.8%)	9(16.4%)		4(19%)	7(33.3%)	
Coronary heart disease, n(%)	N0	29(90.6%)	52(94.5%)	**0.49**	19(90.5%)	16(76.2%)	0.22
	YES	3(9.4%)	3(5.5%)		2(9.5%)	5(23.8%)	
Consciousness state, n(%)	VS	23(71.9%)	16(29.1%)	**<0.001**	17(81.0%)	0(0%)	<0.001
	MCS	9(28.1%)	39(70.9%)		4(19.0%)	21(100%)	
CRS-R score	baseline	6.94 ± 2.61	10.02 ± 2.21	**<0.001**	5.95 ± 1.94	9.14 ± 1.82	<0.001
	6 months	9.28 ± 2.69	15.76 ± 2.06	**<0.001**	8.48 ± 2.04	15.38 ± 1.53	<0.001
	improvement	2.34 ± 0.94	5.75 ± 2.19	**<0.001**	2.52 ± 0.98	6.24 ± 1.73	<0.001

Data are presented as mean ± SD or n (%). *p*-values were calculated using t-test, Mann–Whitney U test, or Chi-square test as appropriate. Bold values indicate statistical significance (*p* < 0.05). CRS-R, Coma Recovery Scale–Revised; GOS-E, Glasgow Outcome Scale–Extended; ICU, intensive care unit; MCS, minimally conscious state; pDoC, prolonged disorders of consciousness; SD, standard deviation; VS/UWS, vegetative state/unresponsive wakefulness syndrome; ICH, intracerebral hemorrhage; TBI, traumatic brain injury; HIE, hypoxic–ischemic encephalopathy.

### Comparative analysis of sex hormone levels between outcome groups

Male subgroup: In univariate comparisons, testosterone levels were higher in the good outcome group (12.64 ± 5.54 nmol/L) compared to the poor outcome group (9.26 ± 5.59 nmol/L, *p* = 0.02), while prolactin levels showed a trend toward being lower in the good outcome group (19.28 [16.04–30.24] ng/mL vs. 26.16 [16.27–34.83] ng/mL, *p* = 0.06). No significant differences were observed in estradiol, LH, or FSH levels between outcome groups (all *p* > 0.05). However, after Bonferroni correction for multiple comparisons (threshold *α* = 0.05/6 ≈ 0.0083), none of the hormone variables remained statistically significant (testosterone: P_bonf = 0.12; prolactin: P_bonf = 0.36). These univariate findings should therefore be interpreted with caution and considered exploratory (Table 2).

**Table 2:** Single-factor analysis of serum sex hormone levels between sex groups by recovery of consciousness status.

Variable	Male				Female			
	Poor outcome (*n* = 33)	Good outcome (*n* = 44)	*p* value	P_bonf value	Poor outcome (*n* = 21)	Good outcome (*n* = 21)	*p* value	P_bonf value
Progesterone (ng/mL)	0.67(0.48–0.81)	0.55(0.41–0.74)	0.11	0.84	0.54 (0.49–0.67)	0.56(0.4–0.79)	0.65	1.00
Testosterone (nmol/L)	9.26 ± 5.59	12.64 ± 5.54	0.02	0.12	0.66(0.38–0.9)	0.72(0.42–1.31)	0.62	1.00
Prolactin (ng/mL)	26.16(16.27–34.83)	19.28(16.04–30.24)	0.06	0.36	29.72(14.29–36.51)	19.48(13.65–30.23)	0.03	0.18
Estradiol (pg/mL)	28.89 ± 13.88	30.81 ± 11.72	0.51	1.00	21.1(12.63–33.42)	19.66 (12.59–24.94)	0.65	1.00
Luteotropichormone (IU/L)	7.88(4.66–10.69)	7.68(4.34–10.60)	0.15	1.00	9.34(2.16, 21.54)	5.54 (1.27–15.84)	0.02	0.12
Follicle-stimulating hormone (IU/L)	7.46(4.89–11.84)	7.32(4.45–9.70)	0.27	1.00	25.02(5.3–38.59)	22.72(9.17–33.67)	0.04	0.24

FSH, follicle-stimulating hormone; GOS-E, Glasgow Outcome Scale–Extended; LH, luteinizing hormone; pDoC, prolonged disorders of consciousness; SD, standard deviation. P_bonf: Bonferroni-corrected *p*-values for multiple comparisons (6 tests per sex group; corrected significance threshold α = 0.05/6 ≈ 0.0083).

Female subgroup: In contrast to males, testosterone levels did not differ significantly between outcome groups (*p* > 0.05) and were markedly lower than male values. Prolactin levels showed a trend toward being lower in the good outcome group (19.48 [13.65–30.23] ng/mL vs. 29.72 [14.29–36.51] ng/mL, *p* = 0.03). Both LH (5.54 [1.27–15.84] IU/L vs. 9.34 [2.16–21.54] IU/L, *p* = 0.02) and FSH (22.72 [9.17–33.67] IU/L vs. 25.02 [5.3–38.59] IU/L, *p* = 0.04) appeared lower in the good outcome group. Estradiol levels showed no significant association with outcomes (*p* > 0.05; Table 2). However, after Bonferroni correction for multiple comparisons (threshold *α* = 0.05/6 ≈ 0.0083), none of these associations remained statistically significant (prolactin: P_bonf = 0.18; LH: P_bonf = 0.12; FSH: P_bonf = 0.24). These univariate findings should be interpreted as exploratory and hypothesis-generating, requiring validation in larger cohorts (Table 2).

### Multivariate analysis of sex hormones predictors for functional outcomes

Multivariate logistic regression was performed to identify independent predictors of 6-month favorable outcomes (GOSE ≥ 4) in male pDoC patients, with adjustment for age, disease duration, and etiology (TBI vs. non-TBI). In the unadjusted analysis, log-transformed testosterone was significantly associated with favorable outcomes (OR = 2.97, 95% CI: 1.29–7.70, *p* = 0.02). However, after Bonferroni correction for multiple comparisons (threshold *α* = 0.05/5 = 0.01), this association did not retain statistical significance (P_bonf = 0.10). Prolactin, age, disease duration, and etiology were not significantly associated with outcomes in either unadjusted or corrected analyses (all *p* > 0.05). The inclusion of etiology (TBI vs. non-TBI) as a covariate did not materially change the effect estimate for testosterone (OR changed from 2.75 to 2.97), and the interaction term between testosterone and etiology was not significant (*p* = 0.68), suggesting that the prognostic value of testosterone is largely independent of injury mechanism. Given the lack of significance after correction for multiple testing, these findings should be interpreted as exploratory and hypothesis-generating, requiring validation in larger prospective cohorts (Table 3).

**Table 3:** Logistic regression analysis of sex hormones predicting patient recovery of consciousness in male.

Variable	OR	95%CI	*p* value	P_bonf value
Age	1.03	(0.99–1.08)	0.84	1.00
Disease duration	0.99	(0.97–1.02)	0.56	1.00
Etiology (TBI vs. non-TBI)	1.92	(0.47–9.19)	0.38	1.00
Testosterone (log-transformed)	2.97	(1.29–7.70)	0.02	0.10
Prolactin (log-transformed)	0.71	(0.33–1.26)	0.31	1.00

CI, confidence interval; TBI, Traumatic Brain Injury; P_bonf: Bonferroni-corrected *p*-values for multiple comparisons (6 tests per sex group; corrected significance threshold α = 0.05/5 = 0.01).

Furthermore, in the multivariate logistic regression analysis adjusting for age, disease duration, and etiology, no female sex hormone was identified as an independent predictor of outcomes (all *p* > 0.05; Table 4). These findings suggest that the univariate associations observed in female patients may be largely explained by confounding factors or limited statistical power due to the small sample size (*n* = 42). The lack of significant associations in both univariate (after correction) and multivariate analyses indicates that the measured sex hormones have limited prognostic value in this female pDoC cohort. However, given the exploratory nature of this study and the small female sample size, these results should be interpreted with caution and warrant further investigation in larger, well-powered studies with standardized endocrine assessments.

**Table 4:** Logistic regression analysis of sex hormones predicting patient recovery of consciousness in female.

Variable	OR	95%CI	*p* value	P_bonf value
Age	1.03	(0.99–1.08)	0.84	1.00
Disease duration	0.99	(0.97–1.02)	0.56	1.00
Etiology (stroke vs. non-strokeI)	0.04	(0.01–1.92)	0.39	1.00
Prolactin	12.78	(1.49–61.48)	0.06	0.56
Luteotropichormone- (log-transformed)	1.11	(0.21–1.26)	0.31	1.00
Follicle-stimulating hormone (log-transformed)	0.64	(0.04–7.28)	0.73	1.00

CI, confidence interval; TBI, Traumatic Brain Injury; P_bonf: Bonferroni-corrected *p*-values for multiple comparisons (6 tests per sex group; corrected significance threshold α = 0.05/6 ≈ 0.0083).

### Prognostic value of testosterone in pDoC patients

**Figure 2:**
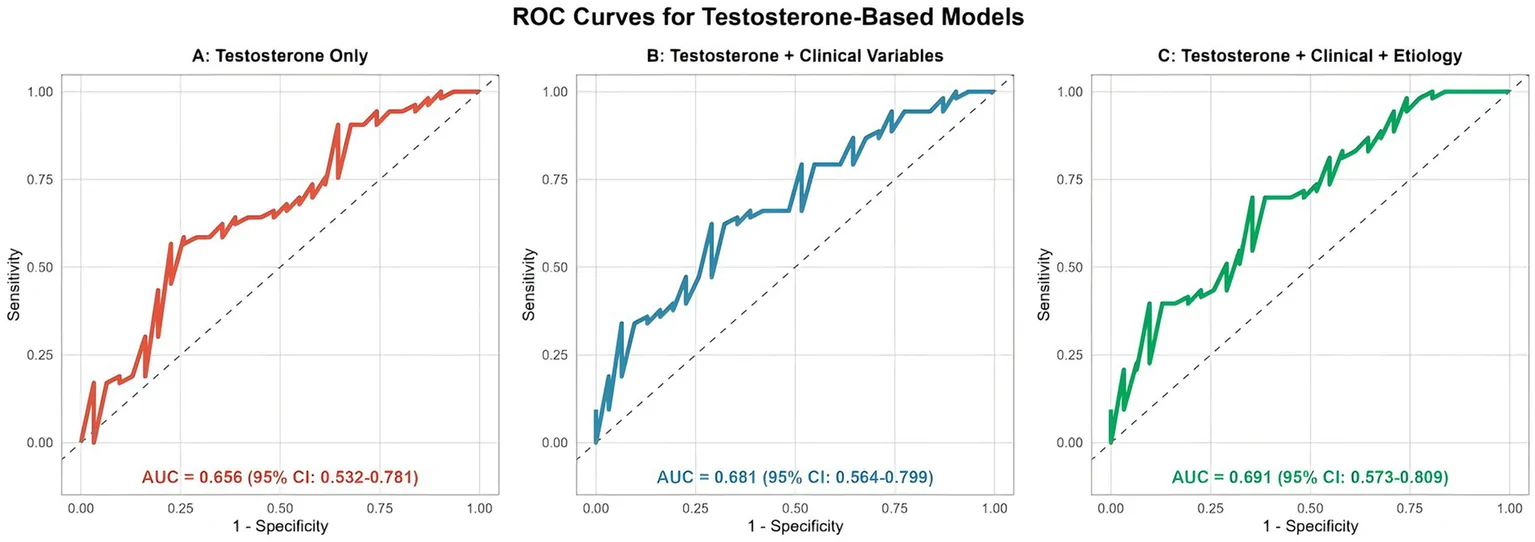
ROC analysis for 6-month outcome prediction in male pDoC patients. Three nested logistic regression models were compared: Model **A** (testosterone alone, red curve) yielded an AUC of 0.656 (95% CI: 0.532–0.781); Model **B** (testosterone + clinical variables: age, disease duration, and craniectomy, blue curve) yielded an AUC of 0.681 (95% CI: 0.564–0.799); Model **C** (testosterone + clinical variables + etiology: TBI vs. non-TBI, green curve) yielded an AUC of 0.691 (95% CI: 0.573–0.809). The DeLong test showed no significant difference between Model B and Model C (*p* = 0.61). AUC, area under the curve; CI, confidence interval; pDoC, prolonged disorders of consciousness; ROC, receiver operating characteristic; TBI, traumatic brain injury.

To evaluate the predictive performance of testosterone and the potential impact of etiology adjustment, we constructed three nested logistic regression models and compared their discriminative ability using ROC analysis (Figure 2). Testosterone alone yielded an AUC of 0.656 (95% CI: 0.532–0.781). After adding clinical variables (age, disease duration and craniectomy), the AUC improved to 0.681 (95% CI: 0.564–0.799). Further adjustment for etiology (TBI vs. non-TBI) resulted in a minimal AUC increase to 0.691 (95% CI: 0.573–0.809), representing a change of only 0.01 from the model without etiology adjustment. The DeLong test showed no significant difference between the models with and without etiology adjustment (*p* = 0.61), indicating that etiology does not substantially affect the predictive performance of the testosterone-based model.

### Spearman’s rank correlation of testosterone and prolactin with CRS-R score in male pDoC patients

**Figure 3:**
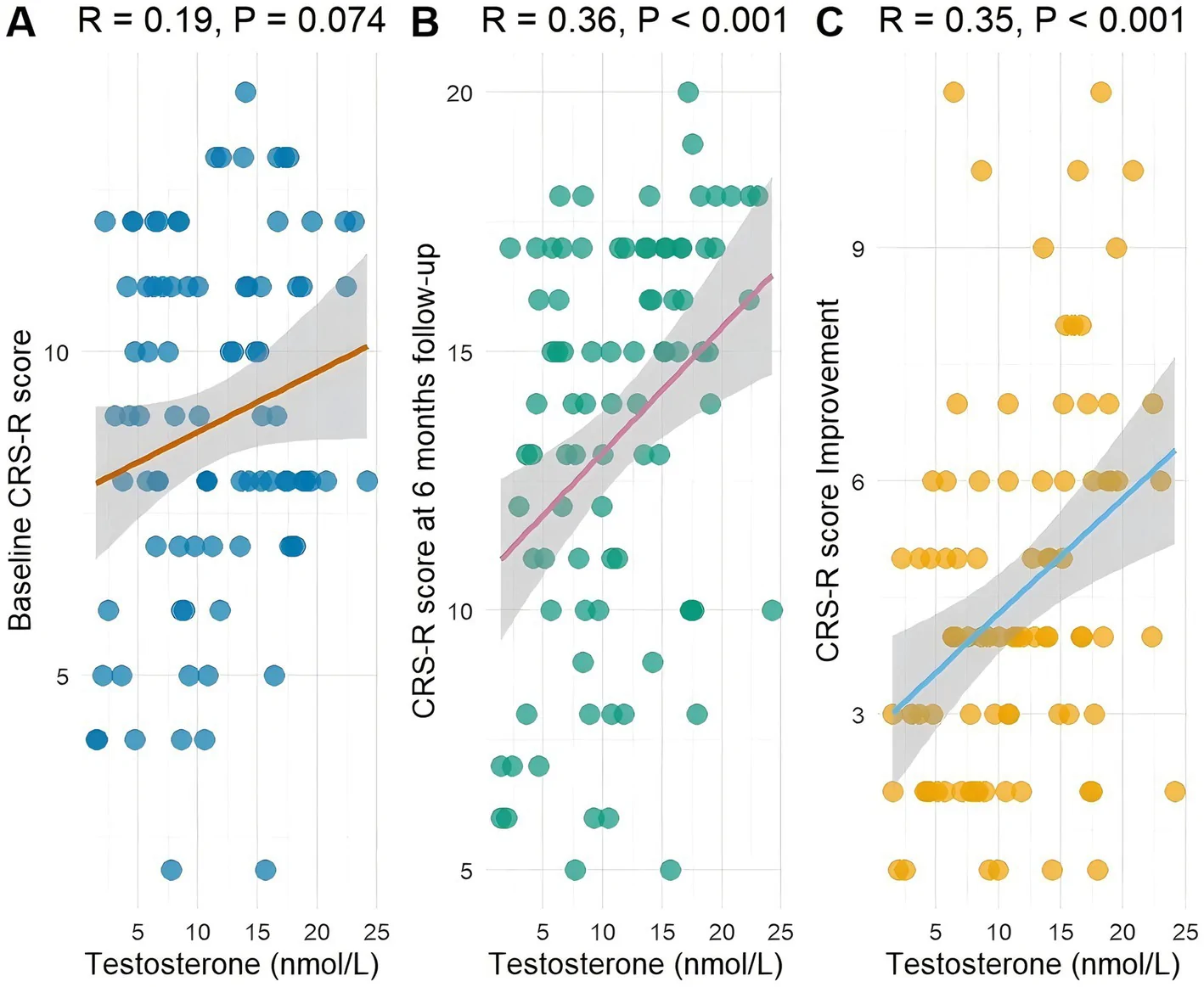
Spearman’s rank correlation of testosterone levels with CRS-R scores in male pDoC patients. Correlation between serum testosterone levels and the CRS-R scores in male patients with pDoC. **(A)** Testosterone levels were not significantly correlated with baseline CRS-R scores (R = 0.19, *p* = 0.07). **(B)** A significant positive correlation was observed between testosterone levels and CRS-R scores at 6-month follow-up (R = 0.36, *p* < 0.001). **(C)** Testosterone levels also showed a significant positive correlation with the improvement in CRS-R scores (ΔCRS-R = 6-month CRS-R– baseline CRS-R; R = 0.35, *p* < 0.001). These findings suggest that higher testosterone levels are associated with better long-term neurological recovery, independent of baseline consciousness severity. CRS-R, Coma Recovery Scale–Revised; pDoC, prolonged disorders of consciousness; ΔCRS-R, change in CRS-R score from baseline to 6 months.

**Figure 4:**
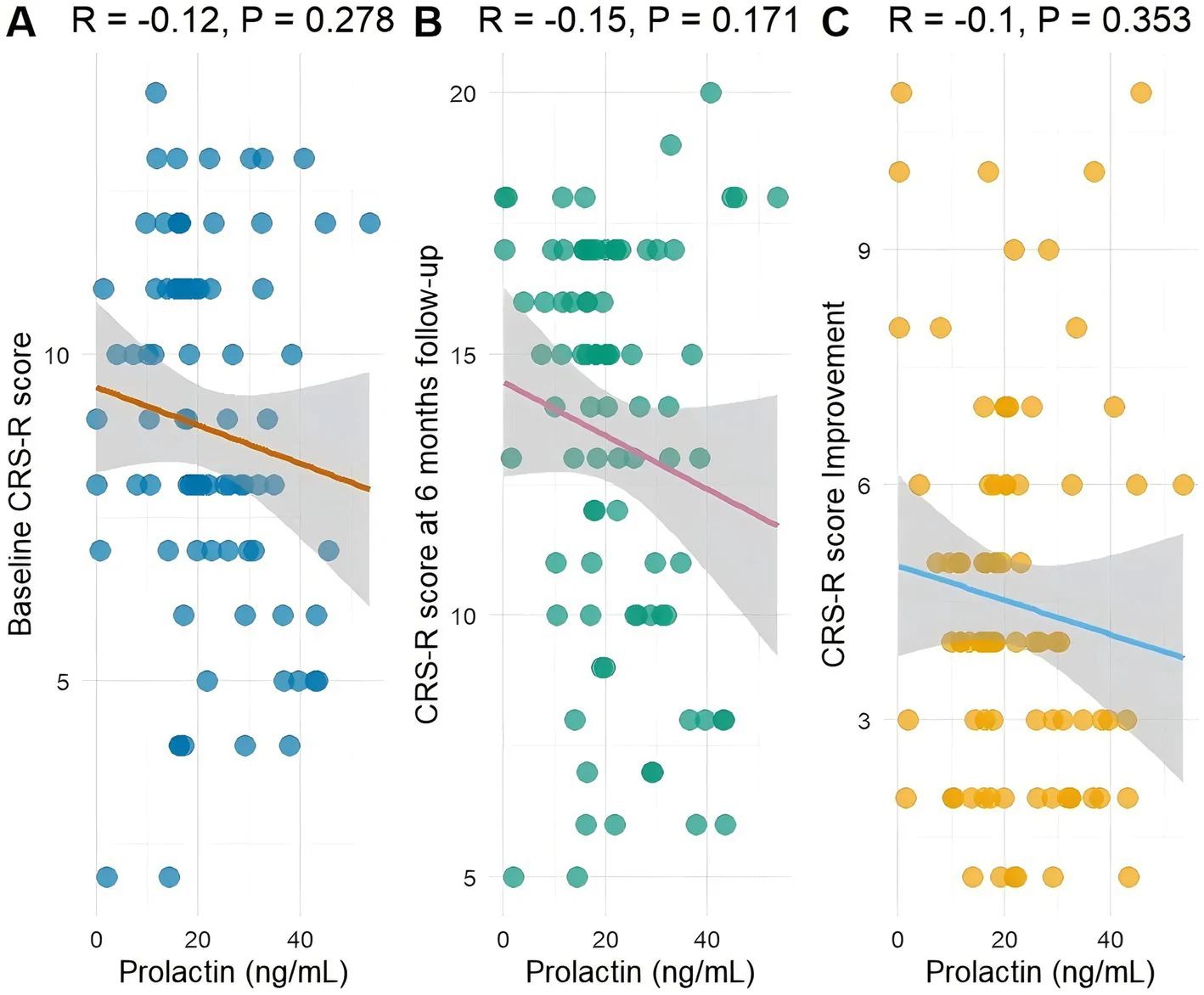
Spearman’s rank correlation of prolactin levels with CRS-R scores in male pDoC patients. Correlation between serum prolactin levels and the CRS-R scores in male patients with pDoC. **(A)** Prolactin levels showed no significant correlation with baseline CRS-R scores (R = −0.12, *p* = 0.28). **(B)** A weak negative correlation was observed between prolactin levels and CRS-R scores at 6-month follow-up (R = −0.15, *p* = 0.017). **(C)** Prolactin levels were not significantly correlated with the improvement in CRS-R scores (ΔCRS-R; R = −0.10, *p* = 0.35). Although prolactin showed univariate associations with functional outcomes, its lack of correlation with CRS-R scores suggests that prolactin may reflect functional capacity rather than the level of consciousness per se in this population. CRS-R, Coma Recovery Scale–Revised; pDoC, prolonged disorders of consciousness; ΔCRS-R, change in CRS-R score from baseline to 6 months.

Testosterone levels, while not correlated with baseline CRS-R scores (R = 0.19, *p* = 0.07), demonstrated significant positive associations with both 6-month CRS-R scores (R = 0.36, *p* < 0.001) and CRS-R scores improvement (ΔCRS-R score; R = 0.35, *p* < 0.001), suggesting that higher testosterone levels predict better long-term recovery trajectories independent of initial impairment severity (Figure 3). In contrast, prolactin showed no clinically meaningful correlations with any CRS-R measures (baseline: R = −0.12, *p* = 0.28; 6-month: R = −0.15, *p* = 0.017; ΔCRS-R: R = −0.1, *p* = 0.35), despite its previously identified prognostic value for functional outcomes (Figure 4).

## Discussion

Current evidence recognizes hypothalamic–pituitary dysfunction as a major complication following both traumatic brain injury and stroke (30–32). However, most research has focused on acute endocrine disturbances, leaving a critical gap in knowledge regarding hormonal changes in pDoC. This clinical challenge is further complicated by the substantial symptom overlap between pituitary dysfunction and typical post-injury manifestations in pDoC patients (33), as well as the lack of routine endocrine assessments in standard care protocols. This oversight has important implications, as previous studies show that early identification and treatment of endocrine deficiencies after brain injury can reduce adverse outcomes and potentially improve recovery (33, 34). Accordingly, from a clinical perspective, accurate prognostic information in pDoC can guide rehabilitation decisions, inform family expectations, and support appropriate resource allocation. The biological relevance of our findings is supported by testosterone’s established neuroprotective effects: promoting axonal regeneration, modulating neuroinflammation, and enhancing synaptic plasticity (35, 36). The positive correlation between testosterone levels and CRS-R improvement over 6 months, though modest, aligns with this mechanistic framework and suggests that testosterone may facilitate gradual neurological reorganization rather than exerting an immediate effect.

pDoC remains a significant therapeutic challenge, with few effective treatments available. Families face profound emotional distress, compounded by difficulties in outcome prediction (37). Consciousness, as the highest integrative function of the cerebral cortex (1), is maintained by complex neural networks, with the ARAS regulating arousal and the hypothalamus governing sleep–wake cycles—core components of consciousness. These neuroanatomical relationships suggest that changes in hypothalamic–pituitary hormones may have predictive value for recovery in pDoC. While the exact mechanisms of hypothalamic–pituitary axis dysfunction after brain injury remain unclear, three primary pathogenic pathways have been proposed (38): (1) primary mechanical injury involving direct structural damage to the hypothalamus or pituitary due to mass effect or increased intracranial pressure; (2) secondary insults such as hypotension, hypoxia, transient stress responses, or delayed autoimmune processes targeting hypothalamic structures; and (3) pharmacologic effects, particularly in patients receiving intensive medical management. Post-traumatic hypothalamic–pituitary–adrenal (HPA) dysfunction usually presents as isolated hormone deficiencies, with complete pituitary failure being rare. The most common endocrine abnormalities include hypogonadism and growth hormone deficiency, followed by adrenocorticotropic hormone and thyroid-stimulating hormone deficiencies (39, 40). Based on these observations, the present study focused on the potential prognostic value of sex hormone levels in pDoC.

Steroid hormones such as testosterone and estradiol are known to exert neuroprotective effects through several mechanisms: stabilizing the blood–brain barrier, reducing cerebral edema, suppressing inflammation, limiting oxidative stress, and decreasing glutamate-induced excitotoxicity (35). Therefore, post-traumatic hypogonadism may impair functional recovery after brain injury. Previous studies have reported significant correlations between low gonadal hormone levels and poor GOSE scores in patients with traumatic brain injury and aneurysmal subarachnoid hemorrhage (41). Bondanelli et al. found that abnormal gonadotropin levels negatively affect cognitive and behavioral recovery, as measured by the Disability Rating Scale (DRS) and Rancho Los Amigos Scale (RLAS) (42). Importantly, the biological response to brain injury shows sexual dimorphism (43). Females tend to be more resilient to neural damage than males, possibly due to the neuroprotective effects of estrogen and progesterone (20). Our findings revealed sex-specific patterns in hormone profiles. In the male pDoC subgroup, testosterone levels were higher in the good-outcome group (*p* = 0.02) with a trend toward lower prolactin levels (*p* = 0.06), while in the female pDoC subgroup, prolactin, FSH, and LH levels appeared lower in the good-outcome group (all *p* < 0.05). However, after Bonferroni correction for multiple comparisons, none of these associations remained statistically significant. These findings suggest that sex hormone fluctuations may be associated with neurological outcomes in a sex-specific manner, though the exploratory nature of these results warrants cautious interpretation and validation in larger cohorts (21, 44).

Several interconnected mechanisms may explain the distinct sex-specific patterns observed. First, testosterone’s neuroprotective effects are mediated through androgen receptor (AR) signaling pathways, which act directly on neuronal ARs in males; in females, circulating testosterone levels are substantially lower, and a significant proportion is aromatized to estradiol, which predominantly activates estrogen receptors rather than ARs (43, 45). Second, endogenous estrogen and progesterone in females may exert overlapping or competing neuroprotective effects that confound the relationship between individual hormones and recovery (24). Third, sexual dimorphism in neuroendocrine responses—including differential patterns of microglial activation following injury—may amplify the prognostic value of androgens in males while diminishing predictive utility in females (43, 46). These sex-specific patterns underscore the importance of stratified analyses in pDoC research.

Multivariate logistic regression identified serum testosterone as a potential predictor of functional recovery in male pDoC patients, though this association did not survive correction for multiple testing. The predictive accuracy was modest (AUC = 0.656), with an optimal cutoff of 11.86 nmol/L, yielding 58.2% sensitivity and 78.1% specificity. These findings are broadly consistent with previous studies (47), although our study identified a higher testosterone threshold and lower diagnostic precision. This discrepancy may reflect differences in the study population, as our cohort was limited to pDoC patients, among whom gradual resolution of central hypogonadism over 3–6 months (48), may reduce the hormone’s predictive sensitivity. Nevertheless, given the exploratory nature of these findings and the lack of significance after correction, these results should be interpreted with caution and require validation in larger cohorts.

Correlation analysis further demonstrated a significant but weak positive association between testosterone levels and 6-month CRS-R score improvements (*p* < 0.001), suggesting that higher androgen levels may enhance recovery potential. Although the exact mechanisms remain unclear, existing evidence supports this association. Testosterone is involved in neural plasticity and its deficiency after traumatic brain injury may negatively affect both short- and long-term cerebral recovery (49).

Clinically, low testosterone is associated with greater injury severity, higher mortality, and lower Glasgow Coma Scale scores, while testosterone replacement has been linked to improved outcomes (50, 51). The neuroactive effects of steroid hormones involve complex modulation of neurotransmission, cortical excitability, and interregional connectivity (52). Functional MRI studies have shown sex-specific mechanisms: ovarian hormones enhance cortico-cortical and cortico-subcortical connectivity, whereas androgens reduce cortical–subcortical integration while strengthening subcortical network coherence (45). Testosterone preferentially activates ventral stream networks during spatial cognition tasks (53),which may explain why testosterone supplementation improves visuospatial and verbal memory in hypogonadal males and patients with Alzheimer’s disease (54). Preclinical models have identified additional neuroprotective effects of testosterone, including reduction of oxidative damage (55), preservation of neural progenitor cell viability under stress (56), and improved memory in aged rats through hippocampal neurotrophic factor regulation (57). Since impaired consciousness results from axonal injury in ascending arousal systems (58), testosterone’s established role in promoting axonal regeneration (36) may further explain its therapeutic relevance in DoC (59). Limited axonal recovery observed in female animals treated with testosterone may be due to peripheral aromatization to estradiol, which does not activate neuronal androgen receptors. Sex-specific neuroendocrine responses to brain injury—affected by menopausal status, parity, and age-related factors^[46]^-may account for the lack of hormone-outcome associations observed in female pDoC patients in this study, consistent with earlier findings (60). These results emphasize the importance of gender-specific approaches in DoC prognosis and management.

In univariate analyses, we observed higher serum prolactin levels in poor-outcome groups across both sexes, with a trend toward significance in males (*p* = 0.06) and a nominally significant difference in females (*p* = 0.03). However, neither of these associations survived Bonferroni correction for multiple comparisons (male: P_bonf = 0.36; female: P_bonf = 0.18). In multivariate logistic regression adjusting for age, disease duration, and etiology, prolactin was not an independent predictor of outcomes in either sex. In males, the OR was 0.71 (95% CI: 0.33–1.26, *p* = 0.31). In females, while a large OR of 12.78 (95% CI: 1.49–61.48) was observed, the association did not reach statistical significance (*p* = 0.06), and the extremely wide confidence interval reflects the limited sample size (*n* = 42) and consequent statistical instability. These findings suggest that the univariate associations observed for prolactin were largely explained by confounding factors and/or limited statistical power, and prolactin does not provide robust independent prognostic information in this cohort. The biological interpretation of these largely null findings warrants consideration. Prolactin is known to be part of the neuro-endocrine-immune response to trauma, with circulating levels often tripling after brain injury, likely due to reduced dopaminergic inhibition from pituitary stalk compression or systemic stress (61, 62). Increased prolactin may disrupt endocrine balance by suppressing GnRH-induced LH secretion, thereby impairing gonadal axis function (63). While preclinical studies have suggested neuroprotective roles for prolactin through glial activation (64), clinical findings remain mixed: some studies have reported that elevated prolactin levels correlate with injury severity and are associated with worse cognitive performance and global outcomes (41, 65, 66), whereas others have not found consistent associations. In our study, prolactin showed no significant correlation with CRS-R scores, 6-month CRS-R scores, or CRS-R improvement in male patients, suggesting that any potential association of prolactin—if present—may be with functional outcomes rather than consciousness recovery per se. However, given the lack of multivariate significance and the instability of the estimate in females, this interpretation remains speculative.

The predictive performance of testosterone (AUC = 0.656 for testosterone alone and 0.691 for the combined model) warrants careful interpretation. On the one hand, the consistent direction of effects across multiple models (ORs > 2.5 in all multivariate analyses) and the routine availability and low cost of serum testosterone testing make it an attractive candidate for clinical integration, particularly in resource-limited settings. On the other hand, the lack of significance after Bonferroni correction, the modest AUC values, and the suboptimal model calibration (Hosmer-Lemeshow *p* = 0.036) temper clinical applicability. We therefore propose that testosterone should not be used in isolation for prognostic decisions but may contribute as an adjunctive marker within a multi-dimensional assessment framework that integrates clinical, neurophysiological, and laboratory parameters. The modest incremental improvement in AUC (from 0.656 for testosterone alone to 0.691 when combined with clinical variables and etiology) supports this adjunctive role, suggesting that while testosterone alone lacks sufficient predictive power, it may enhance the precision of existing clinical assessment tools when integrated with other prognostic indicators.

The lack of statistical significance after multiple comparison correction does not negate the biological relevance of the observed associations but underscores their exploratory, hypothesis-generating nature. The consistent direction of effects—higher testosterone in good-outcome groups across all models—suggests a genuine biological signal warranting further investigation. However, the modest predictive performance and moderate post-hoc power (approximately 68% for the male cohort) indicate that validation in larger, prospective cohorts is essential before clinical translation.

This study has several limitations. First, serum hormone levels were measured at a single timepoint, which may not capture diurnal fluctuations or dynamic changes in endocrine function. Future studies should incorporate serial or continuous sampling to improve assessment accuracy. Second, the relatively small sample size—particularly in the female subgroup (*n* = 42)—limited statistical power and precluded more complex subgroup analyses. This also contributed to suboptimal model calibration and a limited events-per-variable ratio. We acknowledge that our multivariate models did not include baseline CRS-R score or VS/UWS versus MCS status. This was a deliberate decision to avoid overfitting, as testosterone levels showed no significant correlation with baseline CRS-R score in our cohort, suggesting these variables capture distinct aspects of patient status. Nevertheless, we fully recognize that baseline consciousness severity is the strongest established predictor of outcome in pDoC. Its absence from the models therefore constitutes a significant limitation, and our findings should be interpreted with particular caution. Additionally, for female patients, detailed information on menopausal status and menstrual cycle phase at blood sampling was unavailable, precluding adjustment for these physiological confounders. Third, the absence of neurophysiological or neuroimaging data restricted our ability to evaluate the relationships between hormonal profiles, brain network integrity, and recovery potential. Fourth, the post-hoc power of approximately 68% for the primary finding indicates that the study was adequately powered to detect moderate-to-large effects but may have been underpowered to detect smaller effect sizes. Furthermore, the lack of external validation in an independent cohort limits the generalizability of our findings. Future multicenter studies with larger, well-characterized cohorts are needed to validate these exploratory findings and to develop a multidimensional stratification model that integrates clinical severity measures, biomarker profiles, and advanced neuroimaging to improve prognostic precision in pDoC care.

In conclusion, this exploratory study identified sex-specific associations between serum sex hormone levels and functional outcomes in patients with pDoC, although none of these associations remained statistically significant after correction for multiple testing. In male patients, testosterone showed modest, hypothesis-generating associations with neurological recovery, whereas prolactin exhibited only univariate trends that did not persist following multivariate adjustment. No consistent patterns were observed in female patients. Given the modest predictive performance, the limited sample size—particularly in the female subgroup—and the exploratory nature of these findings, testosterone and other sex hormones should not be interpreted as standalone prognostic biomarkers in current clinical practice. Nevertheless, these preliminary observations provide a rationale for further investigating testosterone as a potential neurorestorative marker in males, and underscore the importance of incorporating sex-specific assessment protocols into future pDoC research. Robust validation in larger, prospective, multicenter cohorts will be essential before any clinical translation can be considered.

## Data Availability

The original contributions presented in the study are included in the article/supplementary material, further inquiries can be directed to the corresponding author.

## Glossary

AR: androgen receptor
ARAS: ascending reticular activating system
AUC: area under the curve
CI: confidence interval
CRS-R: Coma Recovery Scale-Revised
CT: computed tomography
DoC: disorders of consciousness
DOCS: Disorders of Consciousness Scale
DRS: Disability Rating Scale
FSH: follicle-stimulating hormone
GOS-E: Glasgow Outcome Scale-Extended
HIE: hypoxic–ischemic encephalopathy
HPA: hypothalamic–pituitary–adrenal
ICH: intracerebral hemorrhage
ICD-10: International Classification of Diseases, Tenth Revision
ICU: intensive care unit
LH: luteinizing hormone
MCS: minimally conscious state
pDoC: prolonged disorders of consciousness
RLAS: Rancho Los Amigos Scale
ROC: receiver operating characteristic
SD: standard deviation
SECONDs: Simplified Evaluation of Consciousness Disorders
SMART: Sensory Modality Assessment and Rehabilitation Technique
TBI: traumatic brain injury
VS/UWS: vegetative state/unresponsive wakefulness syndrome
WHIM: Wessex Head Injury Matrix
